# GTP-dependent run-up of Piezo2-type mechanically activated currents in rat dorsal root ganglion neurons

**DOI:** 10.1186/1756-6606-6-57

**Published:** 2013-12-17

**Authors:** Zhanfeng Jia, Ryo Ikeda, Jennifer Ling, Jianguo G Gu

**Affiliations:** 1 Pain Research Center, Department of Anesthesiology, The University of Cincinnati College of Medicine, PO Box 670531, 231 Albert Sabin Way, Cincinnati, OH 45267, USA; 2 Department of Pharmacology, Hebei Medical University, 361 East Zhongshan Road, Shijiazhuang 050017, China

**Keywords:** Mechanically activated ion channels, Piezo2 ion channels, GTP, G-proteins, Dorsal root ganglion neurons, Pain

## Abstract

Rapidly adapting mechanically activated channels (RA) are expressed in primary afferent neurons and identified as Piezo2 ion channels. We made whole-cell voltage-clamp recordings from cultured dorsal root ganglion (DRG) neurons to study RA channel regulation. RA currents showed gradual increases in current amplitude (current “run-up”) after establishing whole-cell mode when 0.33 mM GTP or 0.33 mM GTPγS was included in the patch pipette internal solution. RA current run-up was also observed in HEK293 cells that heterologously expressed Piezo2 ion channels. No significant RA current run-up was observed in DRG neurons when GTP was omitted from the patch pipette internal solution, when GTP was replaced with 0.33 mM GDP, or when recordings were made under the perforated patch-clamp recording configuration. Our findings revealed a GTP-dependent up-regulation of the function of piezo2 ion channels in DRG neurons.

## Introduction

The ability of animals to detect noxious mechanical stimuli and innocuous touch is essential for life. For example, by detecting noxious mechanical stimuli, one can avoid potentially harmful physical forces. Under pathological conditions such as tissue inflammation and chronic nerve injury, the responses to mechanical stimuli can be greatly enhanced resulting in mechanical allodynia, an exaggerated pain state induced by gentle touch. How mechanical responses are altered under pathological conditions remains to be understood. Several places along the somatosensory system, from peripheral afferent nerve endings to the central sites in the spinal cord and brain, can be involved in the regulation of mechanical responses. Mechanical responses usually start from mechanotransduction at primary afferent nerve endings, a biological process that converts mechanical stimuli into electrical signals. Up-regulation of mechanotransduction at primary afferent nerve endings has been proposed to be a mechanism underlying mechanical allodynia and hyperalgesia. Second messenger systems, including G-protein-coupled receptors, protein kinase A and protein kinase C have been suggested to be involved in up-regulation of mechanotransduction [1,2]. However, mechanotransduction regulation in primary afferent neurons is only partially understood.

Mechanically activated ion channels (MA) have been recognized as the main mechanical transducers in primary afferent nerves. MA currents were first recorded by McCarter et al. [3] from rat dorsal root ganglion (DRG) neurons in culture by using the whole-cell patch-clamp recording technique. Subsequent studies showed that at least two types of whole-cell MA currents could be evoked from DRG neurons, rapidly adapting (RA) and slowly adapting currents (SA) [4]. The RA currents were found to be mediated by nonselective cation channels [5]. It was shown that MA currents were up-regulated via a transcriptional mechanism by the proinflammatory neurotrophin nerve growth factor [1]. Activators of PKC, given in vitro and in vivo, also caused increases in MA currents and behavioral sensitization to mechanical stimulation, respectively [1].

The study of molecular mechanisms of mechanotransduction has recently advanced with the identification of Piezo proteins as MA channels in mammals [6]. Piezo2, one of the two Piezo channels cloned from a mouse neuroblastoma cell line [6], was shown to be expressed in DRG neurons. Knockdown of Piezo2 in DRG neurons specifically reduced RA currents, suggesting that RA currents were mediated by Piezo2 channels [6]. Interestingly, Piezo2 current amplitude was increased and inactivation slowed by bradykinin receptor beta2 activation, and the up-regulation of Piezo2 function was found to be mediated by protein kinase A and protein kinase C [2]. In addition, a role for Piezo2 in EPAC1-dependent mechanical allodynia has been reported recently [7]. Epac1 is a guanine nucleotide exchange factor that activates Rap, a small GTP-binding protein of the Ras family of GTPases [8].

While recording RA currents in cultured DRG neurons, we noticed that RA current amplitude increased over time. It has been known that some ion channels such as voltage-gated Ca^2+^ channels showed increased currents over time during recordings, a phenomenon that is called current run-up [9-12]. RA current run-up has not been reported previously. Therefore, we explored the RA current run-up phenomenon in DRG neurons and identified GTP as a factor involved in the RA current run-up.

## Materials and methods

Adult Sprague Dawley rats (250–350 g, both genders) were used. Animal care and use conformed to National Institutes of Health guidelines for care and use of experimental animals. Experimental protocols were approved by the University of Cincinnati Institutional Animal Care and Use Committee. DRG neuron cultures were prepared as described previously [13]. In brief, rats were deeply anesthetized with isoflurane (Henry Schein, NY) and sacrificed by decapitation. DRGs were rapidly dissected out bilaterally in Leibovitz-15 medium (Mediatech Inc. VA) and incubated for 1 hour at 37°C in minimum essential medium for suspension culture (S-MEM) (Invitrogen, Grand Island, NY) with 0.2% collagenase and 0.5% dispase and then triturated to dissociate neurons. The dissociated DRG neurons were then plated on glass coverslips pre-coated with poly-D-lysine (PDL, 12.5 μg/ml in distilled H_2_O) and laminin (20 μg/ml in Hank’s Buffered Salt Solution HBSS, BD bio-science), and maintained in MEM culture medium (Invitrogen) that also contained nerve growth factor (2.5 S NGF; 10 ng/ml; Roche Molecular Biochemicals, Indianapolis, IN), 5% heat-inactivated horse serum (JRH Biosciences, Lenexa, KS), uridine/5-fluoro-2′-deoxyuridine (10 μM), 8 mg/ml glucose, and 1% vitamin solution (Invitrogen). The cultures were maintained in an incubator at 37°C with a humidified atmosphere of 95% air and 5% CO2. Cells were used after culturing for 3 to 12 days.

Coverslips with cultured neurons were placed in a 0.5-ml microchamber, mounted on an Olympus IX70 inverted microscope (Olympus, USA), and continuously perfused with a normal bath solution at 2 ml/min. The normal bath contained (in mM) 145 NaCl, 5 KCl, 2 MgCl_2_, 2 CaCl_2_, 10 glucose, 10 HEPES, pH 7.3 and osmolarity of 320 mOsm. Unless otherwise indicated, bath solution was maintained at room temperature of 23°C. For conventional whole-cell recordings, the normal patch-clamp internal recording solution contained (in mM) 70 Cs_2_SO_4_, 5 KCl, 2.4 MgCl_2_, 0.5 CaCl_2_, 5 EGTA, 10.0 HEPES, 5.0 Na_2_ATP, 0.33 GTP-Tris salt, pH was adjusted to 7.35 with CsOH and osmolarity was adjusted with sucrose to 320 mOsm. In some conventional whole-cell recording experiments, GTP was omitted or replaced with the same amount of GDP or GTPγS in the internal recording solution. For perforated patch-clamp recording experiments, recording electrodes were filled with internal solution that contained (in mM) 70 Cs_2_SO_4_, 5 KCl, 2.4 MgCl_2_, 0.5 CaCl_2_, 5 EGTA, 10.0 HEPES; pH was adjusted to 7.35 with CsOH and osmolarity was adjusted with sucrose to 320 mOsm; and 60 μg/ml amphotericin B was added just before recordings.

Recordings were performed on small DRG neurons with diameters ranging from 25 to 35 μm. Recording electrode resistance was 3–6 MΩ, and membrane access resistance in the whole-cell configuration was ~10 MΩ and was not compensated. Junction potential between bath and electrode solution was calculated to be 11 mV and was corrected for in the data analysis. Voltage-clamp recordings were performed with cells held at -71 mV (command voltage of -60 mV minus 11 mV for junction potential correction). Signals were recorded with an Axopatch 200B amplifier, filtered at 2 KHz and sampled at 5 kHz using pCLAMP 9.0 (Axon Instruments).

Mechanical stimulation was applied to DRG cell bodies using a heat-polished glass pipette as a probe. The tip size of the probe was approximately 4 μm in diameter. The probe was controlled by a piezo-electric device (Physik Instruments, Auburn, MA) and positioned at an angle of 45° to the surface of the dish. The tip of the probe and the recorded cell were visualized as live images on a monitor throughout the recording. The live images were captured continuously through a CCD camera that was connected to the microscope with 40x objective. The tip was positioned in such a way that a 2 μm movement did not contact the cell, 3 μm had a visible contact but little membrane movement, and a 4 μm stimulus produced an observable membrane deflection. Therefore, tip forward steps of 2 μm and 3 μm were assigned as position -1 and 0 μm, respectively; a 4 μm forward step was recorded as an initial step of 1 μm membrane displacement. The probe was moved at a speed of 0.5 μm/ms. For conventional whole-cell patch-clamp recordings, a fixed mechanical stimulation was applied immediately after breaking into whole-cell mode and then continually applied at an interval of 2 min for up to 30 min. For perforated patch-clamp recordings, a period of 20 min was allowed to achieve good perforation after gigaseal formation, and mechanical stimulation was then applied in the same manner as for conventional whole-cell patch clamp recordings. To ensure the reproducibility of membrane displacement, under visual guidance we corrected any drift just before applying mechanical stimulation in each test. In some experiments, a series of graded membrane displacement steps were applied at 1 μm increments each with 500 ms duration at the beginning and end of the recording. Since membrane displacement might affect the membrane seal and thereby potentially change the access resistance, membrane properties were continually monitored during each recording. This was achieved by applying 5 mV test pulses. Data were discarded if membrane access resistance changed during recordings.

In one set of experiments, Human Embryonic Kidney 293 (HEK293) cells that expressed mouse Piezo2 ion channels were tested. HEK293 cells were grown in Dulbecco’s Modified Eagle Medium containing 10% fetal bovine serum, 50 units/ml penicillin and 50 μg/ml streptomycin. Cells were plated onto 12-mm round glass coverslips placed in 12-well plates and transfected using lipofectamine 2000 (Invitrogen) according to the manufacturer’s instruction. For Piezo2 overexpression experiments, 1 μg/ml of mouse Piezo2 was co-transfected with 0.3 μg /ml GFP to identify transfected cells and cells were recorded 12–24 hours later. Mechanically activated currents in Piezo2-expressing HEK293 cells were recorded under the conventional whole-cell configuration with normal patch-clamp internal recording solution contained 0.33 GTP. Mechanical stimulation was applied in the same fashion as that in the recordings of RA currents in DRG neurons.

Whole-cell recording data were analyzed using Clampfit 9 software. Data are reported as mean ± SEM. Statistical significance (P < 0.05) was assessed by Analysis of Variance (ANOVA, one way) followed by multiple comparison using Student’s t-tests with no corrections [14,15]. Linear regression was applied to assess the trend of MA current changes over time with P < 0.05 being significantly different from the slop of zero (no change of MA currents).

## Results

**Figure 1:**
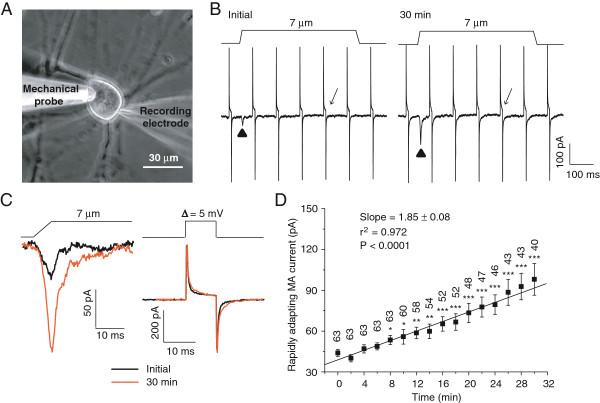
**Run-up of rapidly adapting mechanically activated currents in DRG neurons under the conventional whole-cell patch-clamp configuration. A)** Image shows a DRG neuron with a patch-clamp recording electrode and a mechanical stimulation probe. **B)** Sample traces show the rapidly adapting mechanically activated currents (RA) evoked by a 7-μm membrane displacement. Left panel is the RA current at the initial time (▲ indicated) and right panel the RA current 30 min (▲ indicated) after establishing the whole-cell mode. Membrane displacement is indicated on the top of each trace. The traces also include membrane tests continually conducted at the interval of 100 ms by voltage steps of 5 mV. **C)** Traces are from B at expanded scales. Left, two superimposed traces of RA currents, one at the initial time (solid line) and the other 30 min (orange line) after establishing the whole-cell mode. Right, two superimposed traces of the membrane tests at the initial time (solid line) and 30 min (orange line) after establishing the whole-cell mode. **D)** Summary data of the time course of RA current run-up (n = 40–63 cells). The line in the graph shows linear regression for the mean values of RA currents at different time points. The slope and the coefficient of determination (r^2^) of the linear regression are given in the graph. The P value indicates how significant is the slop (i.e. the trend of change) different from zero (i.e. no change). The number on the top of each symbol indicates the number of cells at each time point. *P < 0.05, **P < 0.01, ***P < 0.001, compared to the initial RA current.

We performed recordings on small-sized DRG neurons. All cells included in the present study had RA currents, and the cells that had no RA currents were excluded from the study. Since RA current amplitude depends on the magnitude of membrane displacement, a small drift of mechanical stimulation probe could introduce errors in RA current measurement. In the present study, we used two methods to ensure the reproducibility of membrane displacement. First, we visualized the tip of the mechanical stimulation probe as a live image on a monitor throughout each experiment and corrected any drift just before applying mechanical stimulation in each test (Figure 1A). Second, in each test, we first measured the mechanical stimulation probe movement step that just produced a visible membrane deflection and then took that step as the initial step of mechanical stimulation. During experiments, we also continually performed membrane tests to monitor membrane resistance and capacitance of recorded cells to ensure that changes in RA currents were confounded by a change of membrane properties during long-time recordings (Figure 1B & C). Under the above conditions and with our normal internal recording solution, we continually tested RA currents at a time interval of 2 min and found that in most cells RA currents gradually increased over time after establishing the whole-cell mode (Figure 1B-D). With a membrane displacement of 7 μm, RA current run-up over time was significant (P < 0.0001) as was determined by linear regression of RA current amplitudes at different time points (Figure 1D). The increases of RA currents reached to a statistically significant level 8 min after whole-cell mode and this RA current run-up was over 2 fold 30 min after establishing whole-cell mode. As shown in Figure 1D, RA currents were 44.0 ± 2.5 pA (n = 63) at initial time, significantly increased to 53.5 ± 3.4 pA (n = 63, P < 0.05) 8 min after establishing whole-cell mode, and further increased to 98.0 ± 11.7 pA (n = 40, P < 0.001) 30 min after establishing whole-cell mode. The RA current decay time constant (τ) was also increased from 4.5 ± 0.3 ms (n = 41) at the initial time after establishing whole-cell mode to 6.8 ± 1.1 ms (n = 32, P < 0.05) 30 min later.

**Figure 2:**
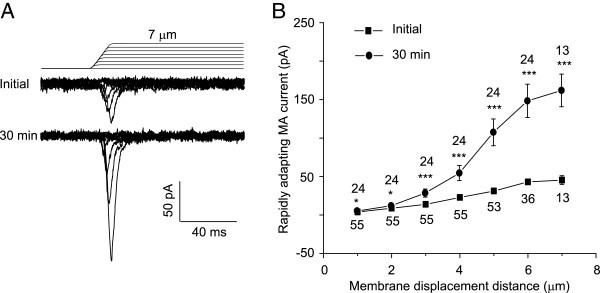
**Run-up of RA currents in DRG neurons at different membrane displacement distances. A)** Two sets of sample traces of RA currents elicited by different membrane displacement steps. Top panel, sample recording at the initial time after establishing the whole-cell mode. Bottom panel, recording 30 min after establishing the whole-cell mode. Membrane displacement steps are indicated above RA current traces. **B)** Summary data of the RA currents at different membrane displacement distances. Solid squares, the initial RA currents immediately after establishing the whole-cell mode (n = 13–55); Solid circles, the RA currents measured 30 min after establishing the whole-cell mode (n = 13–24). The number near each symbol indicates the number of cells tested at each displacement distance. *P < 0.05, ***P < 0.001, comparing the RA currents between initial time and 30 min after the whole-cell mode.

We examined the stimulus–response relationship of RA currents at two time points, the initial time and 30 min after establishing whole-cell mode (Figure 2). At both time points, increasing displacement distances increased RA current amplitude (Figure 2B). When comparing the stimulus–response between the two time points, RA currents at each membrane displacement distance were larger at 30 min than at the initial time (Figure 2B). These indicate that RA run-up occurred at different mechanical stimulation intensities. However, the displacement threshold of RA currents at the initial time after establishing whole-cell mode (2.2 ± 0.3 μm, n = 29) was not significantly different from that measured 30 min later (2.1 ± 0.2 μm, n = 29).

**Figure 3:**
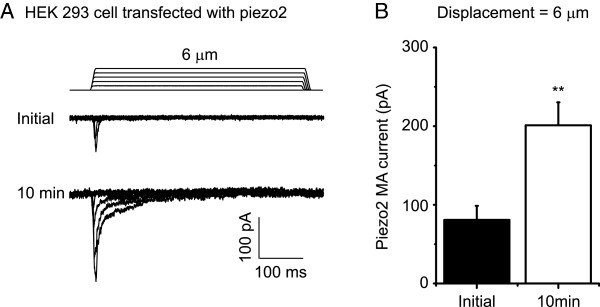
**Run-up of Piezo2 mechanically activated currents in HEK293 cells. A)** Two sets of sample traces of MA currents in a Piezo2-expressing HEK293 cell. Top panel, recording at the initial time after establishing the whole-cell mode. Bottom panel, recording 10 min after establish the whole-cell mode. Membrane displacement steps are indicated above Piezo2 MA current traces. **B)** Summary data of the initial Piezo2 MA currents (n = 6) and the Piezo2 MA currents 10 min after establishing the whole-cell mode (n = 6). *P < 0.05, comparing the Piezo2 MA currents between initial time and 10 min after establishing the whole-cell mode.

We tested if RA currents of Piezo2 ion channels also showed run-up over time under the conventional whole-cell mode. In HEK293 cells that were heterologously expressed Piezo2 ion channels, membrane displacements evoked RA currents (Figure 3A). The decay time constant of Piezo2 currents was 5.4 ±1.7 ms (n = 7), not significantly different from that of RA currents recorded from DRG neurons (4.5 ± 0.3 ms, n = 41). However, Piezo2 currents in HEK293 cells were small initially (80.9 ± 17.8 pA, n = 6) and increased by more than two fold (201.1 ± 29.1 pA, n = 6, P < 0.01) 10 min after establishing the whole-cell mode (Figure 3A & B).

**Figure 4:**
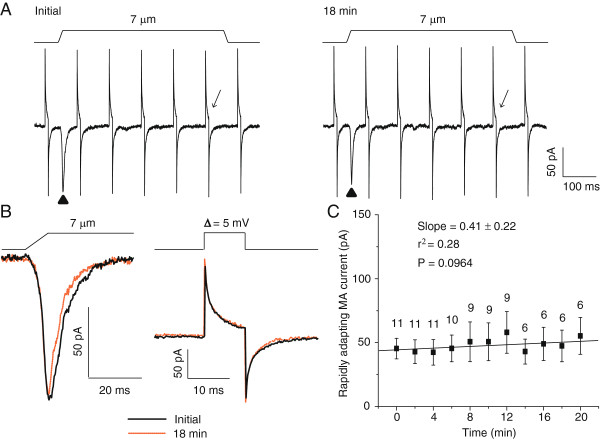
**Lack of RA current run-up in DRG neurons using the perforated patch-clamp recording configuration. A)** Sample traces of RA currents in a DRG neuron measured initially (Left panel) and 18 min later (Right panel). The initial measurement was performed after the patched membrane was perforated to yield a stable access resistance. **B)** The RA currents (left panel) and membrane tests (right panel) in A at expanded scales. The RA currents and membrane tests at initial time are in solid lines and 30 min later in dashed lines. **C)** Summary data of the time course of RA currents measured using the perforated patch-clamp recording technique (n = 6–11). The slope and the coefficient of determination (r^2^) of the linear regression are given in the graph. The P value indicates how significant is the slop (i.e. the trend of change) different from zero (i.e. no change). The number on the top of each symbol indicates the number of cells at each time point.

We used the perforated patch-clamp recording technique to determine whether RA currents would not run-up when intracellular dialysis was prevented. After membrane perforation to reach a stable membrane access resistance (Figure 4A & B), we continually tested RA currents at the time interval of 2 min. As shown in Figure 4, with a membrane displacement distance of 7 μm, there was no tendency of RA current increases (Figure 4C, P = 0.0964 with linear regression). Initial RA currents were 45.4 ± 8.1 pA (n = 11) and were 55.3 ± 14.4 pA (n = 6) 20 min after the initial test. There was no significant difference between the initial RA currents and the RA currents at any time point tested afterward (Figure 4C). The RA currents were stable for up to 20 min, the longest time tested in this set of experiments.

**Figure 5:**
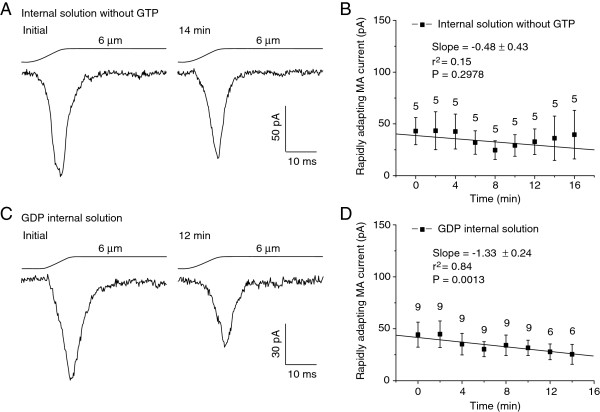
**Lack of RA current run-up in DRG neurons with internal recording solutions that omitted GTP or replaced GTP with GDP. A)** Sample traces of RA currents recorded from a DRG neuron with the internal recording solution that had no GTP. The trace on the left is the initial RA current immediately after establishing the whole-cell mode, and the trace on the right is the RA current recorded 14 min later. Membrane displacement distance was 6 μm. **B)** Summary data (n = 5) of the RA currents at different time points after establishing the whole-cell mode. **C)** Sample traces of RA currents recorded with internal solution that contained 0.33 mM GDP but no GTP. Left panel, initial RA current immediately after establishing the whole-cell mode. Right panel, the RA current recorded 12 min later. Membrane displacement distance was 6 μm. **D)** Summary data (n = 6–9) of the RA currents at different time points after breaking into the whole-cell mode. In both B and D, the slope and the coefficient of determination (r^2^) of the linear regression are given in the graph. The P value indicates how significant is the slop (i.e. the trend of change) different from zero (i.e. no change).

Our normal internal recording solution contained 0.33 mM GTP. We determined whether RA current run-up was dependent on the presence of GTP in our internal recording solution. We first tested this idea by omitting GTP from the internal recording solution and performing recordings under the conventional whole-cell configuration. As shown in Figure 5A & B, RA currents were 39.6 ± 23.5 pA (n = 5) 16 min after whole-cell mode, not significantly different from the initial RA currents of 43.0 ± 13.1 pA (n = 5). Thus, run-up was not observed for up to 16 min when the internal recording solution did not contain GTP (Figure 5D, P = 0.2978 with linear regression). To further validate that GTP in our normally used internal recording solution was required for the RA current run-up, we replaced GTP with the same amount of GDP and then tested RA currents. As shown in Figure 5C & D, RA currents were 44.3 ± 12.0 pA (n = 9) at initial time and 25.4 ± 9.5 pA (n = 6) 14 min after establishing whole-cell mode. Thus, there was no RA current run-up for up to 14 min of the tests with GDP-containing internal recording solution. In fact, there was a tendency of RA current rundown under this condition (Figure 5C & D, P < 0.001 with linear regression).

**Figure 6:**
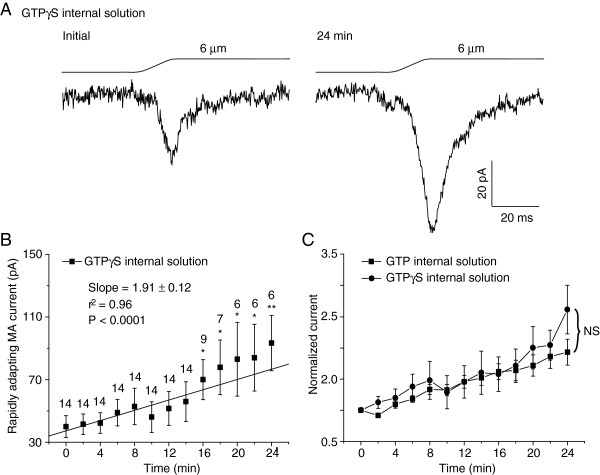
**RA current run-up in DRG neurons with internal recording solution that contained GTPγS. A)** Two sample traces show the whole-cell RA currents recorded from a DRG neuron using internal recording solution that contained 0.33 mM GTPγS. Left, the initial RA current immediately after breaking into whole-cell mode. Right, the RA current 24 min later. Membrane displacement distance was 6 μm. **B)** Time course of RA current run-up with internal recording solution that contained 0.33 mM GTPγS (n = 6–14). The slope and the coefficient of determination (r^2^) of the linear regression are given in the graph. The P value indicates how significant is the slop (i.e. the trend of change) different from zero (i.e. no change). The number on the top of each symbol indicates the number of cells at each time point. * P < 0.05, ** P < 0.01, comparing the initial RA currents to other time points. **C)** Comparison of the RA current run-up between recordings with 0.33 mM GTP (n = 20) and with 0.33 GTPγS (n = 6–14). NS, no significant difference, comparing GTP group and GTPγS group. Normalized currents were calculated from B for GTPγS group and from Figure 1D for GTP group.

We determined if GTP hydrolysis was required for RA current run-up. This was done by replacing GTP with GTPγS, a non-hydrolysable GTP analog, in our internal recording solution. With 0.33 mM GTPγS in our internal recording solution, RA currents showed strong tendency of run-up after establishing whole-cell mode (P < 0.0001 with linear regression). The RA currents were 40.0 ± 7.0 pA (n = 14) at initial time and increased to 93.3 ± 17.6 pA (n = 6, P < 0.01) 24 min after whole-cell mode (Figure 6A & B). The degree of RA run-up with GTPγS was similar to that with GTP in the internal recording solution (Figure 6C).

## Discussion

Mechanotransduction at nociceptive afferent nerve endings normally has high thresholds. An up-regulation of MA channel function would reduce mechanical stimulation thresholds. This is a putative mechanism underlying mechanical hyperalgesia and allodynia. In the present study, we found that RA currents in small-sized DRG neurons were increased over time in a GTP-dependent manner. This is the first report on the role of GTP in regulating RA currents in DRG neurons. The finding may help further understanding of how Piezo2-type MA channels are regulated by intracellular signaling pathways in primary afferent neurons.

We found that RA currents in cultured DRG neurons and Piezo2-medaited RA currents in HEK293 cells increased over time (run-up) during recordings using internal solutions that contained GTP. On the other hand, we did not observe RA current run-up in DRG neurons when GTP was omitted from the internal recording solution. In most previous studies on RA currents in DRG neurons GTP was not included in the internal recording solutions during conventional whole-cell recordings [3,5,7], and RA current run-up was not reported by these previous studies. This seems to be consistent with our finding that RA current run-up was not observed in recordings with the internal solution in which GTP was omitted. RA currents in Piezo2-expressing HEK293 cells and DRG neurons were not reported to have run-up phenomena in two previous studies using internal recording solutions that contained 0.4-0.5 mM GTP [2,6]. It is not clear whether these two studies neglected to mention RA current run-up or because some of their experimental conditions were different from ours. While we showed RA current run-up when conventional whole-cell recordings were performed with internal solutions that contained GTP, We did not observe RA current run-up under the perforated patch-clamp recording configuration. Similarly, in previous studies using the perforated patch-clamp recordings, RA current run-up was also not reported [1,16]. We showed that RA current run-up did not occur when GTP was replaced with GDP, suggesting that RA current run-up was specifically dependent on GTP but not on its metabolite GDP. We found that RA current run-up remained when GTP was replaced with GTPγS, a non-hydrolysable GTP. This suggests that GTP hydrolysis is not required for the RA current run-up.

Intracellular GTP is an important second messenger involved in a number of intracellular signaling pathways. The signaling roles of GTP are largely via different types of GTPases. GTPases are usually active or ‘ON’ when they bind to GTP and inactive or ‘OFF’ when they bind to GDP. It is possible that GTP-dependent run-up of RA currents is mediated by some GTPases, such as G-proteins. Bradykinin receptor beta 2, a G-protein-coupled receptor, has recently been shown to mediate the enhancement of piezo2-type MA currents via protein kinase A and protein kinase C signaling pathways [2]. Enhancement of MA currents by protein kinase C in DRG neurons was thought to be a result of the insertion of new MA channels into the cell membrane [1]. In addition to G-proteins, GTP-bound small GTPases such as Ras, Rho, and Rab families have been shown to regulate ion channel function by affecting ion channel membrane trafficking, protein-protein interactions, and other regulatory mechanisms [17]. GTP has also been known to play important roles in cytoskeleton functions such as direct involvement in microtubule dynamics [18], and a change of cytoskeleton function may affect membrane mechanics and thereby affect MA channel function. In summary, GTP-dependent RA current run-up shown in the present study raises several possibilities for the regulation of piezo2-type MA channels by intracellular signaling pathways.

## Competing interests

The authors declare that they have no competing interests.

## Authors’ contributions

ZJ, RI and JL contributed to experimental design, data acquisition and analysis. JGG contributed to experimental design, data analysis and interpretation. All authors read and approved the final manuscript.
